# Identification of Water Use Strategies at Early Growth Stages in Durum Wheat from Shoot Phenotyping and Physiological Measurements

**DOI:** 10.3389/fpls.2016.01155

**Published:** 2016-08-05

**Authors:** Alireza Nakhforoosh, Thomas Bodewein, Fabio Fiorani, Gernot Bodner

**Affiliations:** ^1^Division of Agronomy, Department of Crop Sciences, University of Natural Resources and Life SciencesVienna, Austria; ^2^Institute for Bio- and Geosciences-2 Plant Sciences, Forschungszentrum JülichJülich, Germany

**Keywords:** drought stress, phenotyping, durum wheat, stomata conductance, water use efficiency

## Abstract

Modern imaging technology provides new approaches to plant phenotyping for traits relevant to crop yield and resource efficiency. Our objective was to investigate water use strategies at early growth stages in durum wheat genetic resources using shoot imaging at the ScreenHouse phenotyping facility combined with physiological measurements. Twelve durum landraces from different pedoclimatic backgrounds were compared to three modern check cultivars in a greenhouse pot experiment under well-watered (75% plant available water, PAW) and drought (25% PAW) conditions. Transpiration rate was analyzed for the underlying main morphological (leaf area duration) and physiological (stomata conductance) factors. Combining both morphological and physiological regulation of transpiration, four distinct water use types were identified. Most landraces had high transpiration rates either due to extensive leaf area (area types) or both large leaf areas together with high stomata conductance (spender types). All modern cultivars were distinguished by high stomata conductance with comparatively compact canopies (conductance types). Only few landraces were water saver types with both small canopy and low stomata conductance. During early growth, genotypes with large leaf area had high dry-matter accumulation under both well-watered and drought conditions compared to genotypes with compact stature. However, high stomata conductance was the basis to achieve high dry matter per unit leaf area, indicating high assimilation capacity as a key for productivity in modern cultivars. We conclude that the identified water use strategies based on early growth shoot phenotyping combined with stomata conductance provide an appropriate framework for targeted selection of distinct pre-breeding material adapted to different types of water limited environments.

## Introduction

Plant breeding for increased water productivity is considered as an essential component to tackle the increased demand for food in view of diminishing water resources for agriculture that are threatening food security over the coming decades (Hall and Richards, 2013; Davies and Bennett, 2015).

Among the three main drought resistance strategies identified by Levitt (1980) in natural ecosystems, traits related to drought escape (Austin et al., 1980; Siddique et al., 1989; Ludlow and Muchow, 1990; Franks, 2011; Isidro et al., 2011) and dehydration avoidance (Richards and Passioura, 1989; Fischer et al., 1998; Rebetzke et al., 2002; Ristic and Jenks, 2002; Gaur et al., 2008; Gowda et al., 2011) have been successfully exploited in breeding for increased crop water productivity. Bodner et al. (2015) described the efficacy of various drought-adaptive traits underlying these strategies in prevailing drought scenarios of rain-fed cropping systems. For supply-driven hydrological regimes with predominant in-season rainfall and intermittent dry spells, water-spending phenotypes with traits sustaining efficient water uptake are most compatible with achieving potential yield (Blum, 2009). Under more severe drought stress in storage-driven environments, where crop growth strongly relies on subsoil moisture stored from off-season rains, early mature phenotypes (drought escape), or water-savers with a balanced use of water throughout the crop cycle are more beneficial to maintain yield.

Progress in yield improvement under suboptimal water availability in cereals has been slower compared to non-water-limited conditions and hampered by the difficulty in selection for plant traits conferring drought resistance under varying drought scenarios in a certain target environment (Austin et al., 1989; Slafer et al., 1994; Richards, 2006; Cattivelli et al., 2008; Tardieu, 2012).

Despite genetic variation for drought resistance in high yielding wheat breeding germplasm, it is still essential to explore the diversity of drought adaptive traits within landraces and wild relatives as potential genetic resources to improve cultivated wheat (Blum et al., 1983; Peleg et al., 2005; Reynolds et al., 2007; Trethowan and Mujeeb-Kazi, 2008).

The developments in genotyping and sequencing in the last decade have resulted in an enormous increase in genomic data. Still genotypic information has to be complemented with the related plant phenotypical traits. Particularly the advance in imaging technology has now led to the development of high-throughput phenotyping platforms to overcome limitations in phenotypic data collection within conventional plant breeding (Passioura, 2012; Kuijken et al., 2015). Current phenotyping platforms provide trait based information on basic (e.g., canopy architecture) and secondary (e.g., chlorophyll fluorescence) plant traits (Fiorani and Schurr, 2013; Granier and Vile, 2014; Fahlgren et al., 2015) including root system architecture (Zhu et al., 2011; Nagel et al., 2012; Downie et al., 2015). Due to the dynamic nature of plant growth, non-destructive imaging provides an important advance to understand responses to environmental factors such as distinct water availability in large numbers of genotypes with comparatively little effort (Berger et al., 2010).

The main aim of our study was to dissect drought resistance strategies within durum wheat landraces from pedoclimatically diverse sites of origin compared to modern cultivars using an imaging-based phenotyping platform (ScreenHouse, IBG2 Plant Sciences, Forschungszentrum Jülich, Germany). Additionally to imaging time courses, the genotypes where characterized physiologically by a single time point gas exchange measurement. Methodologically we hypothesize that differentiation of water use strategies among diverse pre-breeding material requires a combination of whole plant phenotyping with physiological trait information. A second objective was to analyze the relation of water use strategies with crop productivity in the context of early vegetative stage phenotyping. We expect to contribute to improved phenotyping strategies for the detection of crop adaptation to drought by assessing the added knowledge from physiological data to high throughput phenotyping information.

## Materials and methods

### Plant material and sites of origin

The study was performed using 15 durum wheat genotypes (*Triticum turgidum* subsp*. durum*) including 12 landraces and three modern check cultivars originating from contrasting pedoclimatic regions (Table 1).

**Table 1:** **Investigated durum wheat landraces and modern check cultivars and characteristics of the collection sites**.

**Accession number (name)**	**Country**	**Climate^a^**	**Aridity index^b^**	**Longitude (°)**	**Latitude (°)**	**Altitude (m)**	**Soil type**	**Rainfall^c^(mm)**	**ET_0_^c^(mm)**
PI 56238 (Da Terra)	Portugal	Csb	0.61	−9.1	39	120	Cambisol	540.4	523.9
PI 182667 (9923)	Lebanon	Csa	0.40	35.9	33.8	866	Luvisol	517.3	721.4
PI 341521 (Ziraat)	Turkey	Csa	0.60	41	37.4	1060	Luvisol	688.5	655.9
PI 621474 (IWA8608173)^*^	Iran	Dsa	0.36	47	35.7	2080	Xerosol	464.6	573.8
CItr 14610 (ELS 6404-63-7)^*^	Ethiopia	Cwb	0.66	39.0	8.8	2379	Vertisol	710.8	511.2
CItr 14763 (ELS 6404-114-2)^*^	Ethiopia	Aw	0.72	37.3	12.7	2696	Cambisol	920.1	507.8
PI 182699 (B-1)	Syria	BSk	0.30	36.5	32.8	821	Cambisol	351.4	711.0
PI 602420 (96)	Egypt	BWh	0.01	31.6	30.4	22	Fluvisol	17.3	518.1
PI 61105 (6924)	Uzbekistan	BSk	0.08	71.3	40.5	304	Calcisol	7.0	534.1
PI 182113 (S-44)	Pakistan	BWh	0.09	69.0	25.5	0	Yermosol	11.6	443.7
PI 164700 (9127)	India	Aw	0.41	75.2	15.4	648	Nitosol	56.6	423.3
PI 94684 (11BPR)	Armenia	Dfb	0.28	44.5	40.2	1080	Kastanosem	117.7	450.4
cv. NEDA	Iran	Dsa/BSk	0.19	59.6	36.3	1000	Yermosol	248.9	533.2
cv. Floradur	Austria	Cfb	0.84	16.4	48.3	280	Chernozem	240.3	363.4
cv. Levante	Italy	Csa	0.99	11.4	44.5	20	Luvisol	448.6	317.6

a *Based on Koeppen–Geiger climate classification; see Kottek et al. (2006)*.

b *Aridity index classification (UNEP, 1992): AI < 0.05 hyperarid, 0.05 < AI < 0.20 arid, 0.20 < AI < 0.5 semi-arid, 0.5 < AI < 0.65 dry subhumid)*.

c *Refers to rainfall and ET_0_ during the vegetation period of wheat; ET_0_ calculated via Penman-Monteith equation*.

* *The long accession names were shortened as: Ziraat, IWA860, ELS63, ELS114*.

The durum wheat landraces were obtained from the U.S. National Plant Germplasm System (NPGS) which provides precise geographical coordinates of the sites of collection. Germplasm origin covers climatic conditions varying from arid B climates to more temperate C climates with both winter and summer rainfall as well as genotypes from continental D and tropical A climates (Kottek et al., 2006). Pedoclimatic characterization of the sites of origin was done based on data from the FAO weather database and using the New_LocClim software (Grieser et al., 2006) and the harmonized world soil database (Fischer et al., 2008). Table 1 also shows annual rainfall, reference evapotranspiration and their ratio (aridity index; UNEP, 1992) as an indicator of the climatic water balance deficit at the sites of origin of the tested accessions ranging from extremely arid high stress to less stressful sub-humid environments.

### Experimental setup

The genotypes were evaluated in a pot experiment at early vegetative stage for 4 weeks in the PhyTec Experimental Greenhouse at the Institute of Biosciences and Geosciences, Plant Sciences (IBG-2), Forschungszentrum Julich GmbH, Germany (50°54′36″N, 6°24′49″E). The experiment was set up in a factorial completely randomized design with 5 replications for each genotype and treatment. After each measurement the pots were automatically re-randomized via a laser positioning system and a robotic crane to avoid any systematic bias from position within the greenhouse. The fixed factors were genotype (12 landraces and 3 modern cultivars) and water regime (control and drought stress).

The experiment started on June 1st 2014 by visually selecting 20 medium-sized seeds of each genotype and sowing single seeds in plastic germination trays. Uniformly emerged seedlings at the one-leaf stage (BBCH = 11; Lancashire et al., 1991) were then individually transplanted into 5 L pots (23 × 17 cm). Soil substrate was a mixture of peat, sand and pumice (SoMi 513, Dachstauden; Hawita, Vechta, Germany).

For the first week after transplanting, soil moisture level was maintained at field capacity for both the control and stress treatment to ensure optimum establishment of seedlings. Afterwards, all pots were gradually dried down to the predefined moisture levels i.e., 75% (control) and 25% (drought) of plant available water (PAW). PAW was calculated as soil water content difference between field capacity (*h* = −0.01 MPa) and permanent wilting point (*h* = −1.5 MPa) derived from the water retention curve of the soil substrate (Figure S1). The analysis of the soil water retention curve was done in 2013 at the University of Kiel, Germany, Institute of Plant Nutrition and Soil Science. Water content at the respective water potential values was derived from the continuous Van Genuchten retention curve (van Genuchten, 1980). The water content levels of the single pots were then kept constant by automated irrigation after weighing every third day.

Five sensors collected environmental data of the greenhouse (relative humidity, global radiation, temperature) throughout the experiment. In addition to natural light, supplemental illumination was used for 14 h during the experiment. The resulting average light intensity at plant level during the experiment was 442 ± 210 μmol m^−2^ s^−1^.

### Measurements

#### Screenhouse phenotyping platform

The automated ScreenHouse phenotyping platform (*cf*. Supplementary Material for detailed description, Figures S2, S3) provides non-invasive data of plant growth based on projected shoot area by imaging of individual plant shoots three times a week. The platform is equipped with three RGB cameras to acquire different side views of the shoot. Images were analyzed according to the image processing pipeline of the ScreenHouse to extract projected shoot area from images taken from four sides.

Water lost through evapotranspiration was quantified by automatically weighing the individual pots three times a week throughout the experiment. Evapotranspiration rate was then expressed as the amount of water loss per day.

#### Physiological measurements

Gas exchange measurements (stomatal conductance, transpiration, assimilation) were performed 36 DAS (day after sowing) at the central sunlit portion of the youngest fully-expanded leaf of the main stem of three plants per treatment using an infrared gas analyzer (LI-Cor Model 6400, NE, USA) between 0900 and 1300 h (Evans and Santiago, 2014). At this stage (BBCH 29-30) all accessions had sufficiently developed leaf blades for accurate measurement. The single point gas exchange measurement was considered to provide a representative distinction among genotypes having grown under static moisture treatments (drought vs. control) for sufficient time to show steady physiological behavior. An initial light response curve for the modern cultivar Floradur was generated in order to estimate the range of saturating light at which maximum photosynthesis is achieved (Figure S4). This was done by varying light intensity between 0 and 2000 μmol m^−2^ s^−1^ photosynthetic photon flux density (PPFD) provided by a red blue LI-6400-02B light source. Accordingly, a light intensity of 1500 μmol m^−2^ s^−1^ was chosen for measuring gas exchange. Cuvette temperature was set at 20°C, resulting in a steady leaf temperature of 23 ± 0.5°C during the course of measurements. Humidity was set at 60 ± 5% and CO_2_ at 400 μmol mol^−1^.

#### Destructive measurements

Plant phenology was monitored via BBCH growth stages and phyllochron. Tiller number was counted manually prior to harvest on 18th July at BBCH_control_ = 29 and BBCH_stress_ = 30 according to the last developmental growth stage. Harvested plants were separated into leaves and stems and weighed for fresh weight. Subsequently leaf area was measured using a leaf area meter (Li-Cor 3100 Inc., Lincoln, NE). Finally, samples were dried in an oven for 48 h at 80°C to obtain dry matter.

### Statistical analysis

Analysis of variance (ANOVA) was performed on all data using the MIXED procedure in SAS 9.2 (SAS Institute, Inc., Cary, NC). For repeated measures over time (projected area, evapotranspiration) an unstructured covariance model provided the best fit according to the AIC (Akaike Information Criterion). Genotypes and water regimes were treated as fixed effects. Comparison of means was performed using a Tukey *post-hoc* test. Differences among groups of genotypes (i.e., cultivars vs. landraces) and putative water-use strategies were tested by linear contrasts using the CONTRAST statement in the MIXED procedure. Relations among the data were assessed by regression analysis (PROC REG) with stepwise selection for maximum *R*^2^. Slope comparison was performed following the procedure described by Sawand (2012).

## Results

### Water loss by evapotranspiration

The key variable for our analysis of distinctive plant water use among accessions was evapotranspiration rate, i.e., the daily water loss via plant transpiration and soil evaporation.

**Figure 1:**
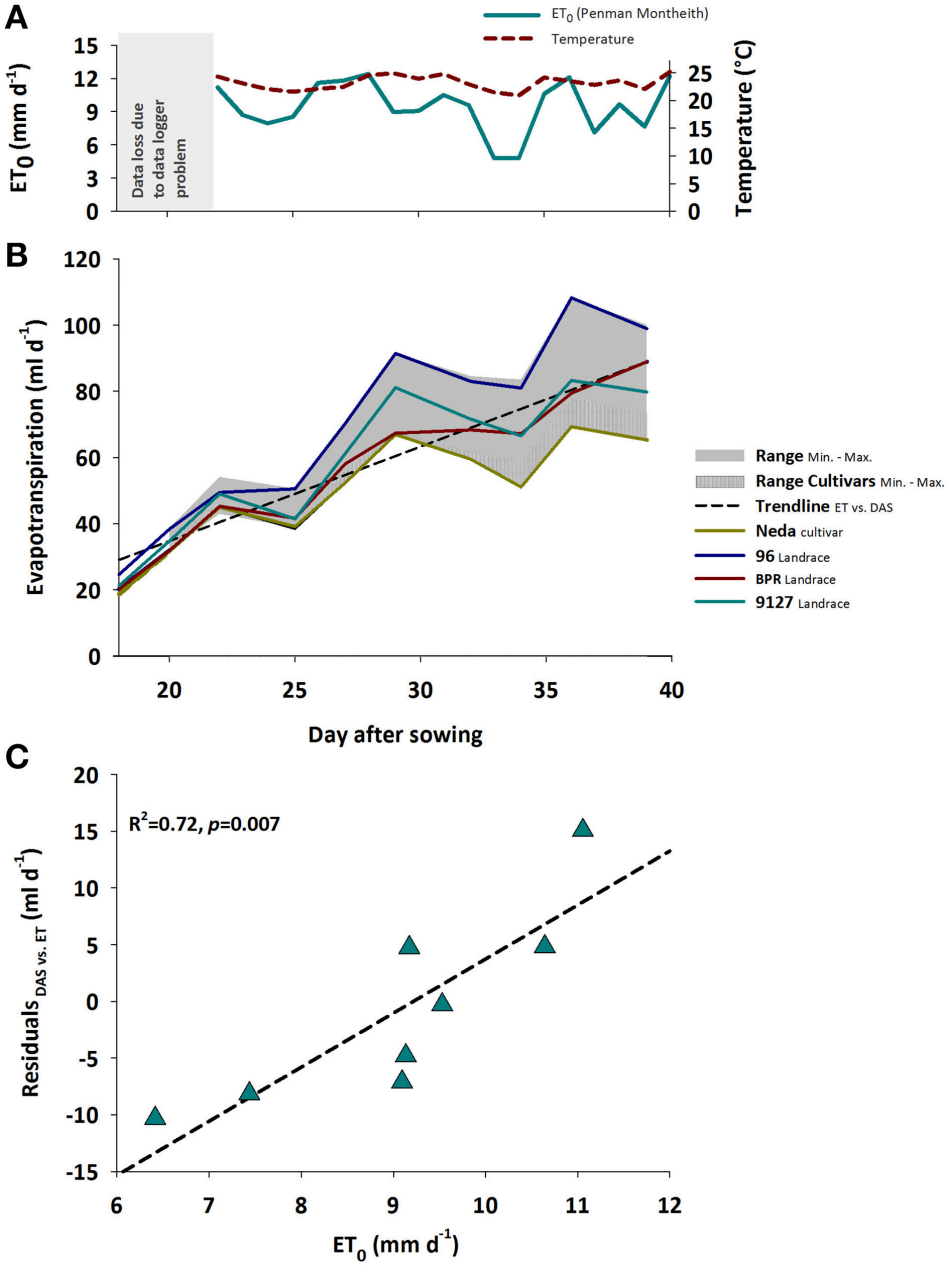
**(A)** Mean temperature and potential (ET_0_); gray area shows period of data logger defect, **(B)** Treatment averaged evapotranspiration rates over time during plant growth showing the range between minimum and maximum for the whole sample and the modern cultivars; separate lines for Neda and 96 representing genotypes with consistently lowest and highest evapotranspiration rates, respectively, as well as for BPR and 9127 exemplifying genotypes with changing ranks over time. Data until day 18 (plant establishment at constant moisture and subsequent drying to defined soil moisture treatments) with predominant evaporation not shown, **(C)** Association of ET_0_ with residuals of the linear regression between evapotranspiration rate and time shown in **(B)**.

Evapotranspiration rate responds to both changing plant properties as well as environmental conditions. Figure 1A shows the daily mean air temperature and reference evapotranspiration (ET_0_, _Penman-Monteith_, Allen et al., 1998) as two key atmospheric variable driving plant growth and development (temperature) as well as water losses (ET_0_).

With increasing leaf development, water loss per day steadily increased. Evapotranspiration rate varied significantly between genotypes and watering treatments, both showing significant interaction with time (*p*_GENxDAS_ < 0.001; *p*_TRTxDAS_ < 0.001). The induced water stress resulted in a decrease of average water loss from 80.2 ml d^−1^ in the control treatment to 44.6 ml d^−1^ in the stress treatment with differences between treatments becoming larger over time.

Figure 1B reveals the significant interaction between genotype × time. Generally evapotranspiration rate over time could be approximated by a linear increase with moderate scattering around this overall trend. Some genotypes like Neda (lowest water-use) and 96 (highest water-use) kept a constant rank over time, while others such as BPR vs. 9127 changed their ranks. Significant differences between Neda and 96 were registered from DAS 25 onwards. BPR with an intermediate water use kept a lower evapotranspiration rate compared to 96 from DAS 25 onwards, while higher rates compared to Neda were found from DAS 32 onwards.

The influence of evaporative demand in the greenhouse was reflected by the scattering of evapotranspiration rate around a linear trend (Figure 1C): the residuals from the linear regression of evapotranspiration rate vs. time were significantly explained (*R*^2^ = 0.72) by reference evapotranspiration (ET_0, *Penman-Monteith*_) calculated from atmospheric data (temperature, radiation, relative humidity) following Allen et al. (1998). As expected the atmospheric demand, approximated by ET_0_, was an important driver of vapor losses from leaf and soil surfaces.

In this experiment no significant interaction between genotype and stress treatment on evapotranspiration rate was found. On average the modern cultivars Neda, Levante, and Floradur had low evapotranspiration rates over time under both control and stress conditions. Similarly, the genotypes ELS 63, 96, and S44 consumed the highest amount of water throughout the experiment, independent of the watering regime (Table 2).

**Table 2:** **Genotypic mean values of water related and plant performance traits of 12 durum wheat landraces and cultivars in response to drought stress**.

**Treatment/genotype**	**DM^a^(g)**	**LAD (cm^2^ d)**	**BBCH**	**Phyllochron (d leaf^−1^)**	**Tillern number (*N*)**	**ET (ml d^−1^)**	**Cum. Transp (mm)**	**SC (mmol m^−2^ s^−1^)**	**PR (μmol m^−2^ s^−1^)**
**CONTROL**									
96	8.5	8533.3	33	3.1	3.4	95.5	48.4	507.8	36.4
6924	5.7	6490.7	34	3.4	2.2	75.7	33.2	637.5	29.5
9127	5.5	6578.1	42	3.7	1.4	83.0	38.7	535.7	34.9
9923	5.0	5716.1	26	4.2	3.2	71.3	31.6	506.6	27.7
BPR	6.9	7276.5	22	5.0	2.8	76.7	34.4	421.0	34.5
B-1	6.0	6732.2	25	3.7	1.4	80.5	37.2	689.0	32.0
Da Terra	6.5	6552.7	31	3.7	2.2	76.5	34.2	387.5	19.5
ELS114	7.5	7944.7	32	3.2	3.0	86.4	41	702.0	31.2
ELS63	7.1	8204.0	34	3.1	2.4	94.9	45.3	829.1	32.1
Floradur	5.2	5582.7	35	2.9	1.8	74.0	32.1	762.6	32.8
IWA860	6.7	7246.2	22	5.0	3.0	81.9	39.2	439.1	25.5
Levante	4.6	5094.8	28	3.7	1.2	71.6	31.8	560.1	26.2
NEDA	4.7	5033.4	31	3.2	1.8	66.6	26.5	828.8	36.5
S-44	7.6	7943.8	19	3.7	1.6	88.4	43	344.1	18.0
Ziraat	6.8	7720.6	23	3.4	3.4	79.4	36.5	242.8	12.3
s.e.d.	0.5	443.4	2.1	0.6	0.7	2.1	2.5	71.9	1.6
**DROUGHT**									
96	4.9	6189.3	35	2.6	0.8	54.2	26.9	490.6	31.5
6924	2.5	3337.6	35	3.7	1.0	36.8	14.4	602.8	31.0
9127	2.9	4131	46	3.7	0.8	42.8	19.3	524.8	37.0
9923	3.5	4631.3	19	6.1	1.6	45.9	21.1	413.8	25.5
BPR	4.2	5117.9	22	5.5	2.6	45.2	20.1	392.0	27.5
B-1	3.4	4849.3	26	4.2	2.8	43.9	19.4	481.7	28.2
Da Terra	3.5	4037.2	31	3.7	0.8	42.3	18	259.9	17.0
ELS114	3.8	5290.2	34	3.9	1.4	45.9	20.6	483.0	27.9
ELS63	4.4	5741.4	35	3.1	1.2	54.8	27.2	715.8	35.5
Floradur	3.0	3817.5	35	2.8	1.6	40.8	16.9	587.6	33.8
IWA860	3.6	4455.8	23	7.9	2.6	40.1	17.4	366.9	27.0
Levante	2.5	3236.7	32	3.1	0.6	39.6	16.1	527.4	30.3
NEDA	2.9	3516.9	35	3.2	1.4	40.2	16.7	664.7	35.5
S-44	4.4	5386.9	25	3.9	1.6	49.8	23.7	284.4	17.3
Ziraat	4.5	5504.0	24	6.1	3.6	47.2	21.5	202.3	12.7
s.e.d.^b^	0.3	318.3	2.4	0.8	0.5	1.3	1.6	76.2	2.2
Genotype	< 0.0001^c^	< 0.0001	< 0.0001	< 0.0001	0.0075	< 0.0001	< 0.0001	< 0.0001	< 0.0001
Treatment	< 0.0001	< 0.0001	< 0.4946	0.0111	0.0039	< 0.0001	< 0.0001	0.003	0.278
G × T	0.101	0.512	< 0.5079	0.0890	0.3420	0.166	0.138	0.812	0.219

a *DM, Dry-matter; LAD, Projected leaf area duration; ET, Evapotranspiration rate; Cum. Transp, Cumulative transpiration; SC, Stomatal conductance; PR, photosynthetic rate*.

b *s.e.d., Standard error of differences*.

c *p-value for genotype, treatment, and their interaction (G × T)*.

#### Leaf area influence

Beyond the environmental influence shown in Figure 1C, our key interest is on relevant plant properties underlying different evapotranspiration rates and how they differ among the investigated genotypes. In our study we hypothesized that the plant influence is related to both canopy traits, particularly leaf area, and physiological traits.

Leaf area over time was obtained from the projected shoot area measured in the ScreenHouse. Figure S5 shows the tight relation between projected shoot area and destructively measured leaf area at the end of the experiment. Thus the imaged shoot area provided an acceptable estimate for leaf area at early vegetative stage during the duration of the experiment. Genotype differences in leaf area development were captured using leaf area duration (LAD; Table 2) as an integrative leaf trait for the transpiring surface area over time. Water deficit significantly decreased LAD of all genotypes (control: 6843.3 cm^2^ d; stress: 4616.2 cm^2^ d). Again the genotype × treatment interaction was not significant indicating a common response pattern among the genotypes. Modern cultivars were at the lower end of LAD under both moisture treatments, while the Ethiopian landraces ELS63 and ELS114, and the Egyptian 96 were at the upper end with most extensive canopies. Applying linear contrasts within a one-way ANOVA demonstrated that the group of modern cultivars had an average lower leaf area compared to the group of landraces (cultivars, 4380.3 cm^2^ d; landraces, 6067.1 cm^2^ d; *p* < 0.001).

**Figure 2:**
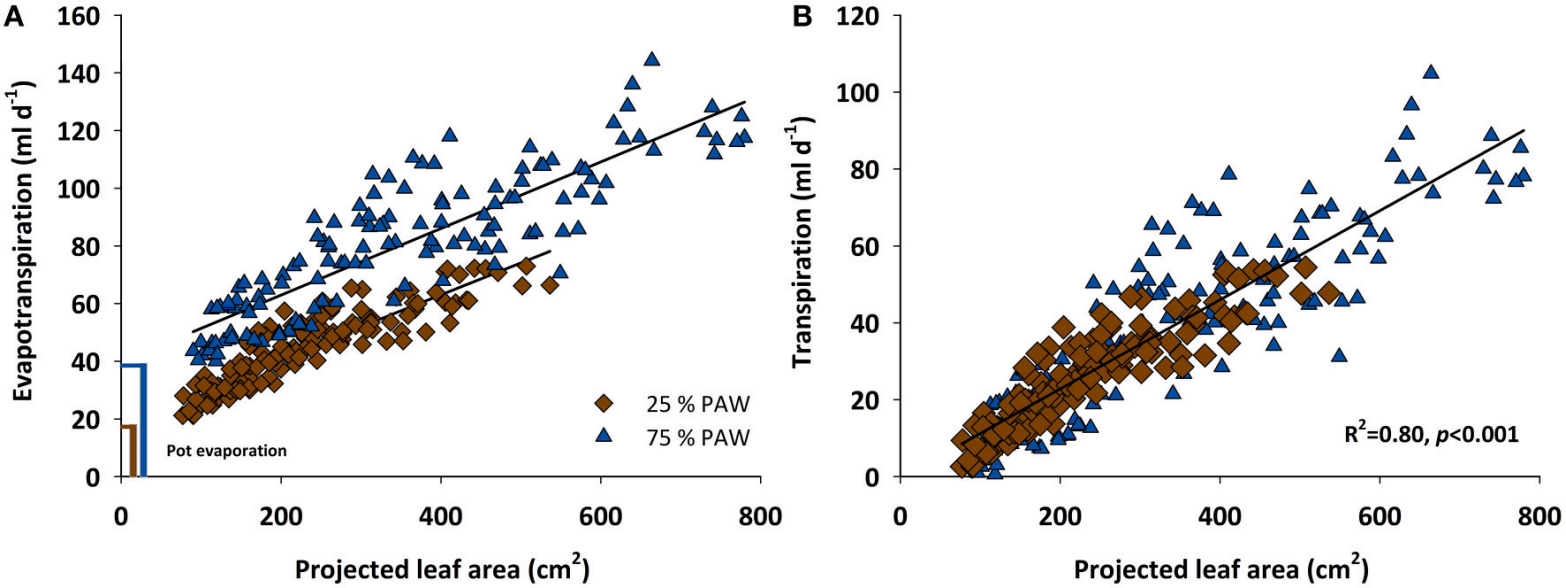
**Scattered diagram showing relationship between (A) evapotranspiration rate and (B) transpiration rate vs. the projected leaf area of 12 durum wheat landraces and 3 cultivars in response to drought stress during the vegetative growth (BBCH = 10–39)**. PAW, plant available water.

From DAS 18 onwards the increasing evapotranspiration rates showed strong association with the expanding leaf surface of the genotypes (Figure 2A). Linear regressions of evapotranspiration rate vs. leaf area followed identical slopes under control and stress conditions. Differences among the two watering regimes were mainly evident from the size of the intercept in Figure 2A, which represents the different amount of evaporation from the pot surface. Although pots were covered using white reflective plastic granules (2–3 mm diameter; Macomass AG, Aschaffenburg, Germany) in order to minimize evaporation, still there was a considerable gaseous water loss from the soil surface. After subtracting soil evaporation (control, 39.4 ml d^−1^; stress, 18.6 ml d^−1^) the linear relation between transpiration rate and leaf area followed the same trend line in stress and control water regimes (Figure 2B).

**Figure 3:**
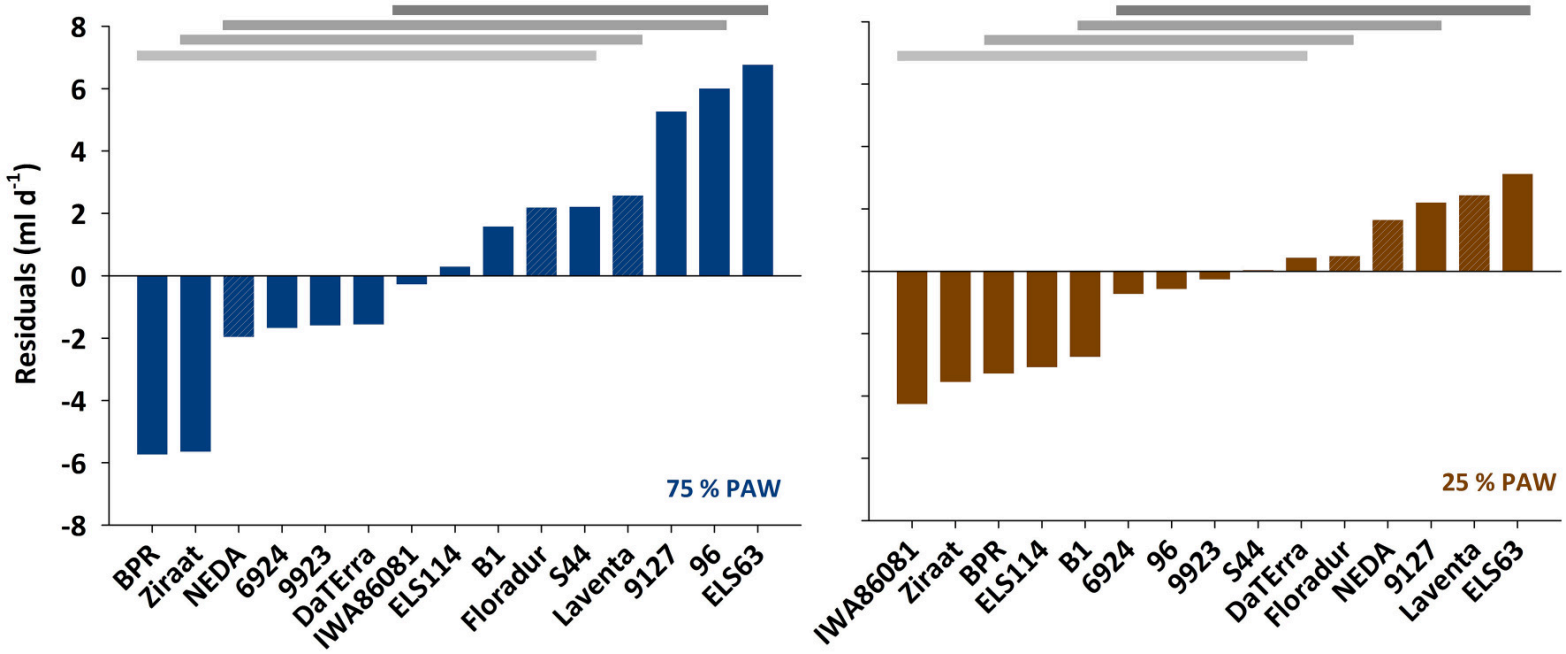
**Residuals from the linear regression between transpiration rate and leaf area under two different soil moisture regimes**. PAW, plant available water. Means with common horizontal bars at the top are not significantly different (Tukey's test, *p* < 0.05).

In spite of the tight relation between leaf area and transpiration rate, the scattering around the regression line in Figure 2 suggests that there were additional mechanisms determining a given transpiration value. Indeed when inspecting the residuals of the linear regression between transpiration rate vs. leaf area, these were not just random errors but contained a significant genotype × treatment effect (Figure 3). ELS63 for example had a much higher transpiration rate than expected from its leaf area under both moisture regimes, (i.e., a highly positive value of residuals) compared to most other genotypes. Ziraat (control + stress), BPR (control), and IWA86081 (stress) on the contrary had a substantially lower transpiration rate as predicted from their leaf areas (i.e., negative residuals), again differing from most other genotypes.

#### Stomata influence

Besides the morphological traits related to canopy architecture, physiological regulation essentially determines the amount of plant water loss. Here we focus on stomata conductance as an additional explanatory trait of physiological type.

Stomatal conductance varied significantly between genotypes and moisture treatments with no significant interaction between main effects (Table 2). Stress-induced reduction in stomatal conductance was 16% compared to the control treatment (559.6 vs. 466.5 mmol m^−2^ s^−1^). The modern cultivars all had above average stomatal conductance with highest values observed in Floradur and Neda. Some landraces like Ziraat and S44 showed constitutively very low conductance independent of the water regime. Also for stomatal conductance linear contrasts supported the assumption that modern cultivars as a group could be distinguished from the group of landraces (cultivars, 655.2 mmol m^−2^ s^−1^; landraces, 477.5 mmol m^−2^ s^−1^; *p* < 0.001).

**Figure 4:**
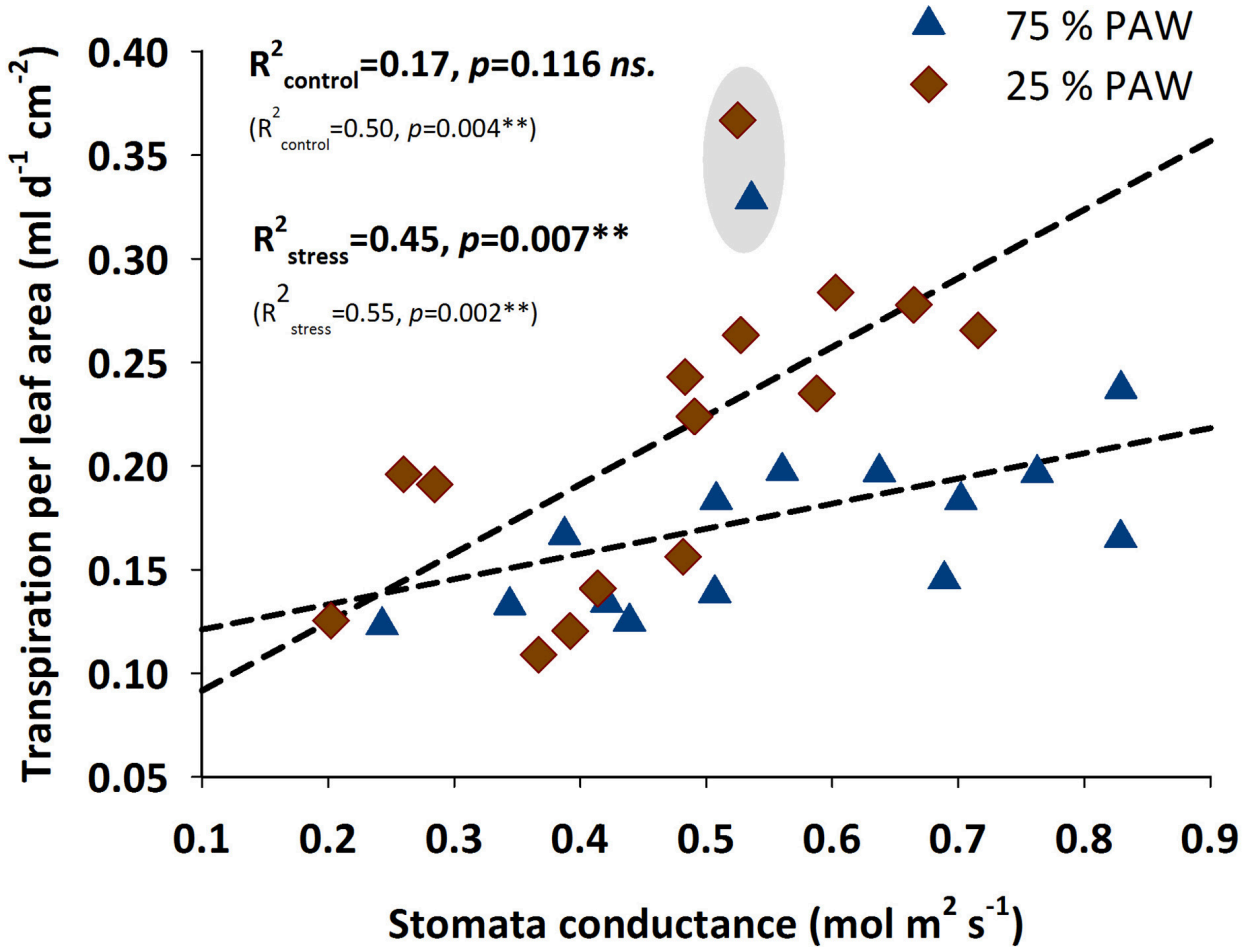
**Relationship between water transpired per unit leaf area and stomatal conductance of durum wheat landraces and cultivars in response to drought stress**. *R*^2^ (coefficient of determination) and *p*-values with (bold) and without (in parenthesis) accession 9127 considered as an outlier from the relation.

For testing the relation of transpiration rate with stomatal conductance (Figure 4), we standardized transpiration rate per unit leaf area (using the directly measured leaf area after harvest). Under stress conditions stomatal conductance clearly played an important regulatory role determining the amount of transpiration per unit leaf area. For well-watered conditions this relation was not significant. However, when excluding accession 9127 as a putative strong outlier, also for the control treatment a significant linear regression would be obtained, with still the slope of the regression being significantly (*p* = 0.008) lower compared to the stress treatment.

### Water use types

Multiple linear regression with leaf area duration and stomata conductance as dependent variables explained 89% of the observed variation in transpiration rate. Both variables significantly contributed to the model.

**Figure 5:**
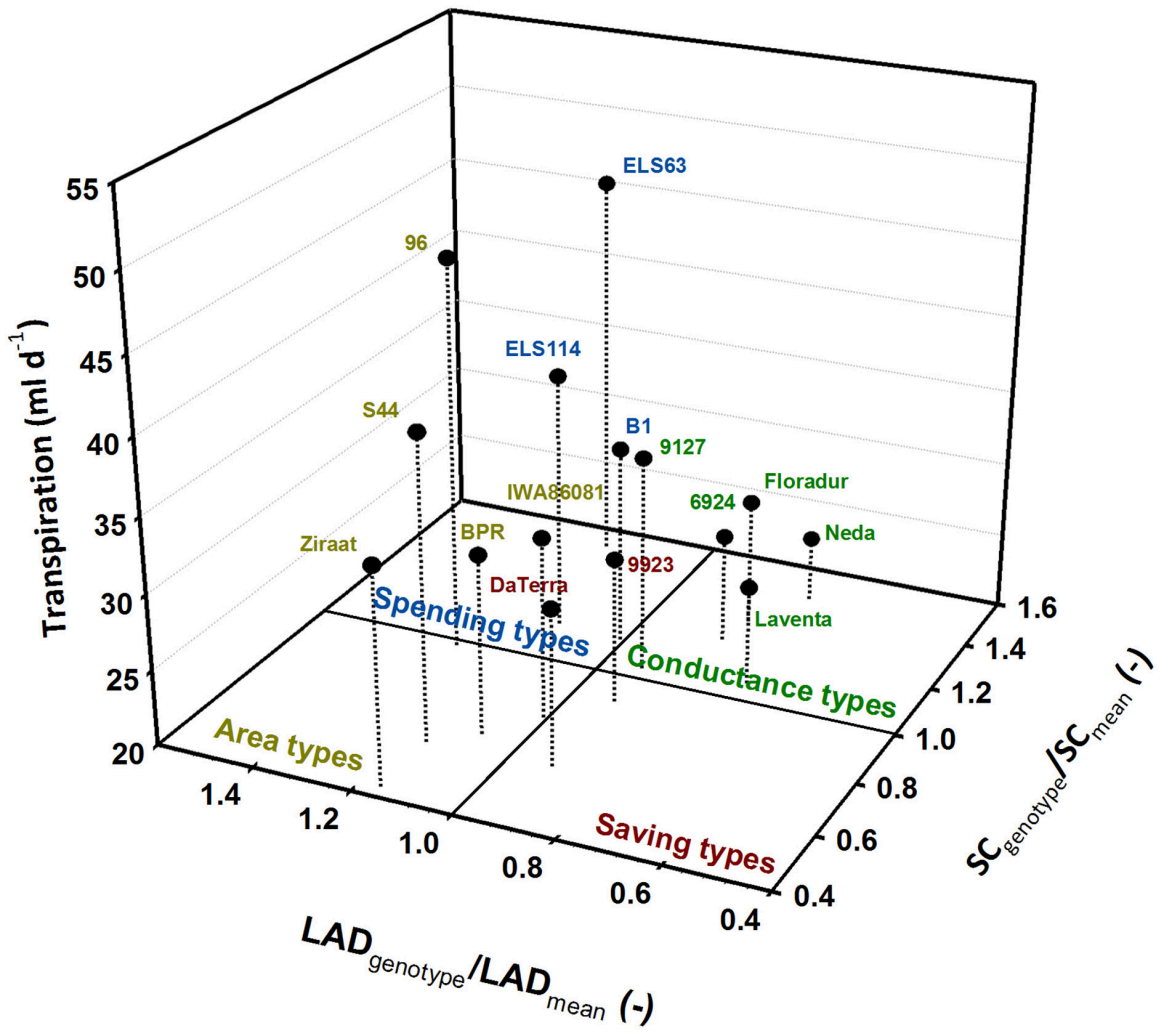
**Differentiation of durum wheat landraces and cultivars to four water use types; traits (LAD, leaf area duration; SC, stomatal conductance) are standardized by their means**.

Plotting the values of these traits standardized by their treatment means allocates the genotypes to four groups. This visualization identifies four different water use types (Figure 5) which can be hypothesized to distinguish the single genotypes according to their specific trait combinations.

Genotypes with leaf area duration and stomatal conductance higher than the average represent water-spending types (ELS114, ELS63, B1). Genotypes with high leaf area duration but lower conductance than average were defined as area types (96, BPR, IWA86081, S44, Ziraat). Few genotypes had both lower than average leaf area duration and stomatal conductance, constituting a group of water-saving types (9923, Da Terra). All modern cultivars (Floradur, Levante, Neda) as well as two landraces (6924, 9127) were characterized by lower leaf area duration and higher stomatal conductance than average. We denominated this group as conductance types.

**Figure 6:**
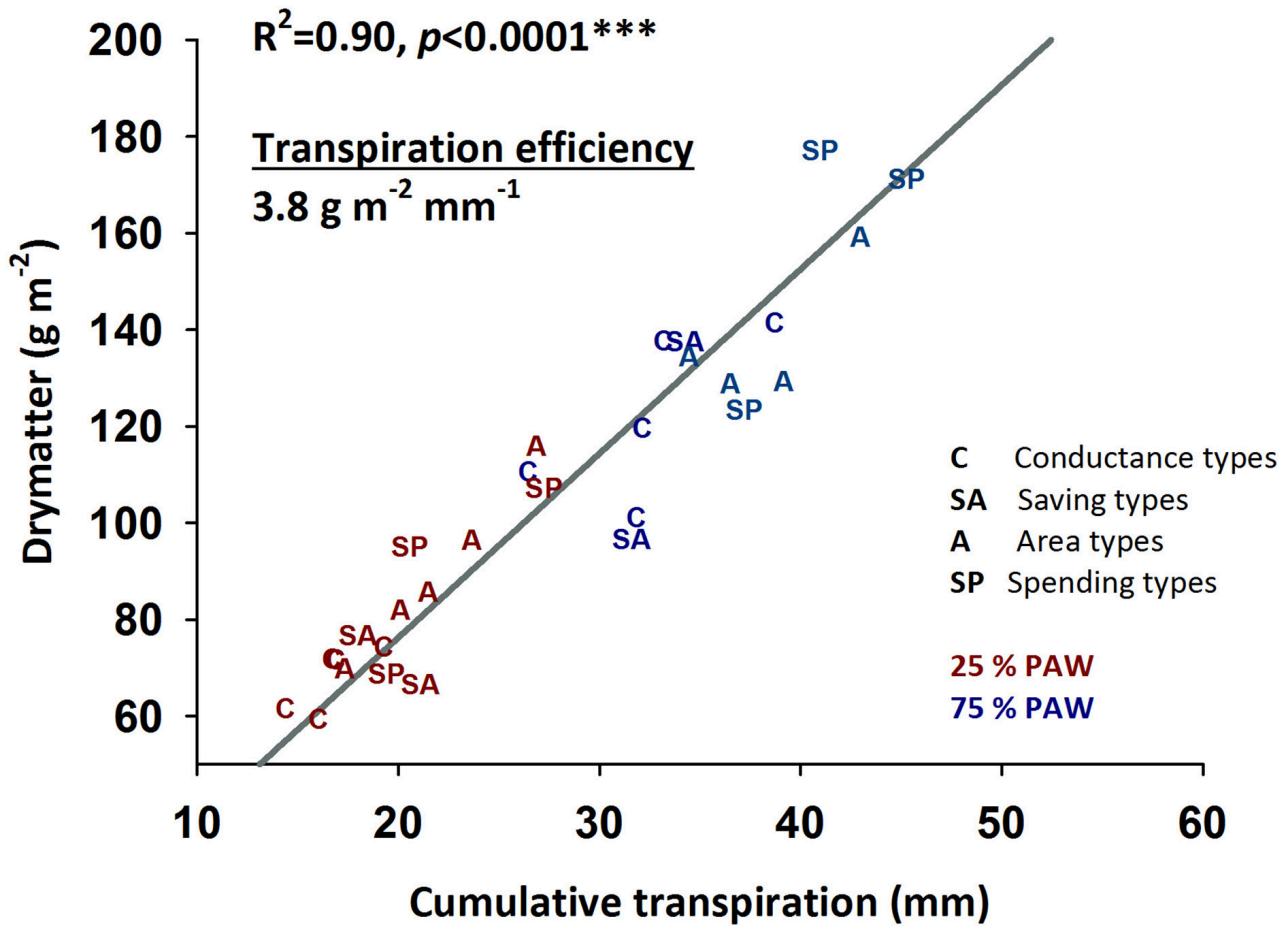
**Relationship between cumulative water transpired during early vegetative growth and accumulated dry matter (transpiration efficiency) of durum wheat landraces and cultivars under well-watered and drought stress conditions**.

The groups identified from this analysis (Figure 5) were then analyzed by linear contrasts in a one-way ANOVA with treatment (TRT; control, stress), trait (TRAIT; leaf area duration, stomata conductance) and water use type (TYPE; spender, saver, area, conductance) as fixed effects. TYPE (*p* < 0.001) and the interaction of TYPE × TRAIT (*p* < 0.001) were significant, while all other effects were not significant. Thus the water use types emerging from Figure 6 were stable over the two moisture treatments. Comparison of means confirmed the differences determining the four water use types (Table 3).

**Table 3:** **Average transpiration rate (TR) and underlying traits (LAD leaf area duration, SC stomata conductance; relative values standardized by treatment mean) of the four distinctive water use types identified from Figure 5**.

**Type**	**LAD_relative_ (−)**	**SC_relative_ (−)**	**TR (ml d^−1^)**
Saver	0.92aA	0.79aA	30.0a
Area	1.13bA	0.68aB	36.9b
Conductance	0.82aA	1.26bB	28.2a
Spender	1.19bA	1.24bA	38.7b

*Lower-case letters refer to the comparison of water use types in each trait, while upper-case letters refer to the comparison among the underlying traits for each water use type*.

Average transpiration rate differed significantly among water-spenders and area-types vs. water-savers and conductance types. Thus, transpiration rate alone would have suggested only two distinctive groups, while consideration of underlying morphological and physiological traits identified four distinctive water use strategies among genotypes.

### Dry matter accumulation

Having identified distinct water use strategies, the main question in a crop production context is their implication for growth potential. In case of the ScreenHouse phenotyping platform where genotypes are observed in their early stages only, this refers to vegetative (from germination to pre-flowering stage) dry matter accumulation.

Dry matter showed significant genotype and treatment main effects without significant interaction. Exposure of genotypes to drought stress led to a strong decrease in accumulated dry matter (6.3 vs. 3.6 g; Table 2) which was a common response for all genotypes.

Differences in treatment averaged dry matter accumulation of the four water use types resembled their differences in transpiration rate: Water-spending types (5.3 g) and area types (5.8 g) significantly contrasted with water-saving types (4.6 g) and conductance types (4.0 g).

We notice here that the tested accessions also differed in phenology (BBCH, phyllochron) and tillering (*cf*. Table 2). However we did not find strong influence of these traits on both transpiration (control: $R_{\text{BBCH}}^{2}$ < 0.01, *p* = 0.929; $R_{\text{tiller}}^{2}$ = 0.09, *p* = 0.269; stress: $R_{\text{BBCH}}^{2}$ = 0.02, *p* = 0.611; $R_{\text{tiller}}^{2}$ < 0.01, *p* = 0.917) and dry matter (control: $R_{\text{BBCH}}^{2}$ = 0.05, *p* = 0.442; $R_{\text{tiller}}^{2}$ = 0.27, *p* = 0.045; stress: $R_{\text{BBCH}}^{2}$ = 0.18, *p* = 0.117; $R_{\text{tiller}}^{2}$ = 0.13, *p* = 0.179). Still an indirect influence of these traits on dry matter accumulation can be assumed via their clear relation to final leaf area (control: $R_{\text{BBCH}}^{2}$ = 0.68, *p* < 0.001; $R_{\text{tiller}}^{2}$ = 0.28, *p* = 0.041; stress: $R_{\text{BBCH}}^{2}$ = 0.72, *p* < 0.001; $R_{\text{tiller}}^{2}$ = 0.66, *p* < 0.001).

#### Leaf area and stomata influences

Again we assume that dry matter is a product of leaf area duration (light interception) and stomata conductance (CO_2_ assimilation and transpiration; both traits *cf*. Table 2). Similar to transpiration rate, LAD was strongly related to dry matter ($R_{\text{control}}^{2}$ = 0.76, *p* < 0.001; $R_{\text{stress}}^{2}$ = 0.78, *p* < 0.001), while on a per unit leaf area basis a significant contribution of stomatal conductance could be shown, particularly under stress conditions ($R_{\text{control}}^{2}$ = 0.26, *p* = 0.0544; $R_{\text{stress}}^{2}$ = 0.42, *p* = 0.009). The higher *R*^2^ for this relation under stress conditions underlines that in case of water limitation stomata opening constrains the potential of assimilation, while under well-watered conditions other photosynthetic traits determine the actual amount of assimilated CO_2_. This is also underlined by the relation between assimilation (PR; Table 2) and stomata conductance from gas exchange measurements: under stress stomata opening conditions assimilation with an $R_{\text{stress}}^{2}$ of 0.84 (*p* < 0.001), while under well-watered conditions the $R_{\text{control}}^{2}$ for this relation was only 0.41 (*p* = 0.010). Finally also the lack of significance for the soil moisture treatment effect on assimilation rate (28.6 vs. 27.9 μmol m^−2^ s^−1^; *p* = 0.2897), contrary to what was found for stomata conductance (559.6 vs. 466.5 mmol m^−2^ s^−1^; *p* = 0.003) points to a changing role of the single processes co-limiting assimilation under variable water supply conditions.

A multiple regression model with both LAD and SC as key variables explained 83.0% (control; *p*_*LAD*_ < 0.001, *p*_*SC*_ = 0.0421) and 81.5% (stress; *p*_*LAD*_ < 0.001, *p*_*SC*_ = 0.131) of the total variance in dry matter. Although, stomata opening was more strongly related to dry matter produced per unit leaf area under stress, its relative weight compared to leaf area duration for overall dry matter accumulation was less at limited water supply.

We finally mention that both explanatory traits, leaf area and stomata conductance, are partially also related to differences in plant phenology, i.e., early genotypes had a comparatively lower final leaf area ($R_{\text{control}}^{2}$ = 0.68, *p* < 0.0001; $R_{\text{stress}}^{2}$ = 0.72, *p* < 0.0001) with higher stomata conductance ($R_{\text{control}}^{2}$ = 0.46, *p* = 0.0054; $R_{\text{stress}}^{2}$ = 0.44, *p* = 0.0070) compared to later ones.

#### Transpiration efficiency

Crop productivity in water limiting environments is frequently assessed by transpiration efficiency revealing the amount of dry matter produced per unit water transpired (Figure 6). In our experiment an average transpiration efficiency of 3.8 g m^−2^ mm^−1^ (slope of the linear regression in Figure 6) was obtained over the two moisture treatments. Transpiration efficiency varied between genotypes and water regimes (control, 3.7 g m^−2^ mm^−1^; stress, 4.0 g m^−2^ mm^−1^), but no interaction of genotype × treatment was observed (*p* = 0.316).

The four groups of genotypes with distinctive water use strategies did not show significant differences in their average transpiration efficiencies: genotypes with lower water use (savers, conductance types) achieved low total dry matter, while those showing higher water consumption also accumulated highest dry matter over the duration of the experiment.

## Discussion

### Phenotyping for drought resistance

The objective of phenotyping is to support the selection process for superior cultivars by specific trait information (Passioura, 2012). Here used image based shoot phenotyping combined with physiological measurements to identify the diverse mechanisms of drought response within a pre-breeding sample of durum wheat genetic resources compared to modern cultivars. Different frameworks have been used to reveal relevant traits for drought resistance. For example Passioura (1977) defined yield formation under drought as the product of water use, water use efficiency and harvest index. Reynolds and Tuberosa (2008) identified traits to be selected for in order to improve each of these single components. Another framework of drought resistance frequently used in breeding studies follows Levitt (1980), distinguishing between drought escape, dehydration tolerance, and dehydration avoidance. Relevant traits for each of the single strategies have been elaborated by Farooq et al. (2009) and Blum (2011).

ScreenHouse captures shoot traits of plants during the early vegetative stage until increasing leaf overlap impedes accurate inference on canopy growth via image analysis only. Our observations demonstrate that canopy growth and architecture essentially drive the two functions of interest in drought resistance: dry matter accumulation and transpiration water losses. Water saving vs. spending as two distinct strategies of dehydration avoidance is first of all related to differences in the transpiring leaf area over time. Drought escape mostly refers to the timing of stress sensitive stages in relation to stress occurrence in a given environment, i.e., addressing stress effects on reproductive processes beyond the phenological development of plants in this experiment. However, also early vigor influences the dynamics of canopy closure and thereby light and water use efficiency. Loel et al. (2014) for example associate historic yield increases in sugar beet with more vigorous early growth resulting in quicker canopy closure and thereby improved light interception. Furthermore, early vigor is also relevant for lower soil evaporation losses by shading (Richards, 1996) and, in some climates, growth under less water demanding conditions due to lower saturation deficit of the atmosphere during a longer part of the crop cycle. These consequences of phenological differentiation can be readily captured by the ScreenHouse imaging platform, thereby providing important information to infer on potential drought escape advantages.

Pot experiments require a sound understanding of experimental conditions (Poorter et al., 2012). The imposed hydrology in pots is the basis for a sound interpretation of phenotyping observations toward plant breeding. In the current study a constantly lower water content compared to a well-watered control was used. Passioura (2012) showed that this might discriminate against water savers using less water during irrigation intervals, while spenders with high water consumption are favored by receiving more water: e.g., the total irrigation amount required to maintain the pre-set moisture level in the stress treatment (25% PAW) was 28% less for the saving type Da Terra compared to the spending type ELS63. The saving types on the contrary would have profited from their lower water demand in case of an experimental setup stopping irrigation at a certain point. In such a case lower transpiration by reduced leaf area could be expected to result in higher stress tolerance compared with water spenders (i.e., less reduction of dry matter) because of delayed exhaustion of soil moisture and therefore later stomata closure and wilting.

We did not observe interactions between genotype and watering treatment in most traits; i.e., superior genotypes under well-watered conditions were also superior under drought, although the imposed stress regime clearly reduced growth (−42% average dry matter reduction). Using dry matter accumulation as selection criteria, water spenders generally outperformed all genotypes with a small leaf area. The lower transpiring surface and the related limitation of water demand did not provide any advantage under the regular rewetting regime in terms of balancing water availability over longer time. The smaller leaf area however limited light interception and thereby the potential for dry matter accumulation under both moisture treatments. In natural environments with severe and prolonged drought, dehydration avoidance via traits limiting water demand (saving types Da Terra and 9923) still may be beneficial. They ensure better water availability for grain filling and yield formation (Condon et al., 2004; Monneveux et al., 2005; Mori et al., 2011). On the other hand water savers might tend to suboptimal use of available soil water under mild to moderate water deficits, thereby limiting the capacity for photosynthesis and biomass accumulation (Rebetzke et al., 2002). In such conditions water spenders (accessions ELS114, ELS63, B1) would profit from their high leaf area and stomatal conductance, implying high carbon gains and growth rates such as observed in our phenotyping experiment. In rain fed agro-ecosystems the yield advantage of spenders however frequently depends on root system traits that confer optimized water uptake to buffer intermittent stress periods (Peleg et al., 2005). Therefore, root system information is of particular interest to study possible allometric relations between shoot and root traits (e.g., Siddique et al., 1990) that sustain the productivity advantage of landraces with extensive leaf area we would expect from early stage phenotyping.

### Morphological and physiological drivers for water use and dry matter accumulation

A frequently used breeding target is transpiration efficiency, i.e., the amount of dry matter per unit water transpired. Still the outcomes of targeting improved transpiration efficiency are contradictory (Araus et al., 2002; Condon et al., 2002; Blum, 2005). Transpiration efficiency is a function of whole plant morphology, i.e., the leaf area driving transpiration and light interception, and leaf scale physiology, i.e., gas exchange at the stomata level and photosynthetic capacity (Steduto et al., 2007). Our data showed an increase in transpiration efficiency under dry conditions. This is explained by the stronger reduction of transpiration compared to assimilation when stomata close: assimilation decreased by only −2.9% with a −15.9% reduction in stomatal conductance under stress. For transpiration stomata are the main resistance, while CO_2_ transport is also dependent on mesophyll resistance; thus the relative response of assimilation to stomata conductance is less compared to the response of transpiration (e.g., Farquhar and Sharkey, 1982; Barbour et al., 2010). It is worth mentioning that both relevant components of transpiration efficiency, leaf area, and stomata conductance, are related also to distinct phenology among accessions. Similar to our results, Condon et al. (2002) found that early genotypes with high crop growth rate are associated with higher stomata conductance, while their leaf area expansion is constraint by the shorter duration of growth stages.

During early growing stages morphological traits are the main limiting factor, i.e., the low leaf surface acts as the predominant constraint to assimilation (via light interception) and transpiration. Only at later stages, when leaf area is fully developed and light interception is at maximum, other traits related to physiological efficiency at leaf level can become predominant constraints (Richards, 2000). Morphological differences in plant canopies are therefore the principal driver during an early stage phenotyping experiment. This implies that the two components of transpiration efficiency, dry matter and transpiration, are reduced proportionally. The importance of the physiological response, resulting in a proportionally lower decrease of dry matter compared to transpiration, is still subordinated. During later stages of plant growth, however, when leaf area is sufficient for maximum light interception, physiological efficiency at the single leaf scale can become essential for enhanced plant productivity. Udayakumar et al. (1998) argue that a yield advantage might be achieved from higher transpiration efficiency when the underlying reason is superior photosynthetic capacity rather than lower stomata conductance. Steduto et al. (2007) however consider the variability in photosynthetic capacity as limited under comparative nutrient and water status of photosynthetic tissues within a given species and during vegetative growth. Therefore, Blum (2009) argued against transpiration efficiency as a selection target, except for high stress conditions where stomata mediated water saving ensures reproductive success at relatively low yield levels.

Particularly for early stage phenotyping, transpiration efficiency does not provide the best selection criterion. Differentiation among accessions is clearly less compared to dry matter and transpiration alone: coefficients of variation under well-watered conditions were 10.7% for transpiration efficiency, 15.9% for transpiration, and 18.4% for dry matter respectively, while under stress they were 9.0, 18.7, and 21.1%. The common leaf area dependence of both parameters underlying transpiration efficiency hides existing variability among accessions for each single process.

**Figure 7:**
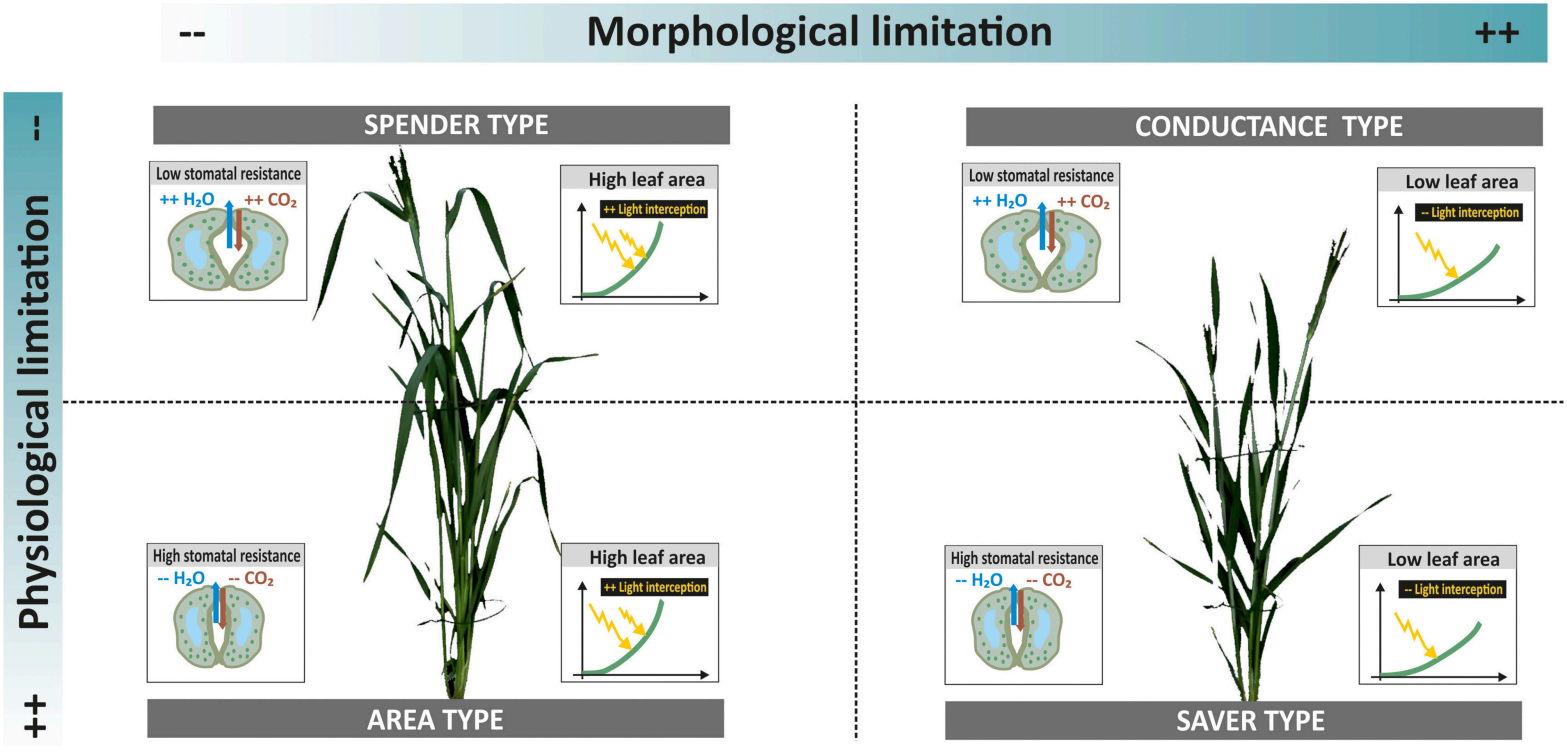
**Different types of accessions in relation to water use and dry matter accumulation. Limitations can be via morphological and physiological traits**. Their combination results in four strategies with different implications for plant productivity under drought. Example plants from ScreenHouse RGB imaging (**left side** spender type landrace ELS 114; **right side** conductance type cultivar Neda).

In order to go beyond the expected dominance of the leaf area influence on both dry matter and transpiration during early stage phenotyping, it is necessary to assess traits and processes per unit of leaf area. This reveals elements of plant productivity others than those mediated by extensive canopies, particularly those related to physiological regulation via stomata conductance. Thereby a more differentiated picture of water use and dry matter accumulation strategies among accessions can be obtained. Figure 7 resumes the distinction among accessions when combining morphological phenotyping data (leaf area) and physiological measurements (stomata conductance).

Two thirds of landraces belonged to types characterized by extensive canopies (area and spending types), while all three modern cultivars were in the group of capacitance types with compact canopy and high stomatal conductance. Only two landraces where identified as water savers with low leaf area and restricted stomata conductance. This suggests that landraces tend to produce an extensive vegetative canopy to ensure successful establishment, while probably investing excessively in vegetative biomass at the cost of grains. The important role of grain sink limitation for cereal yield advance has been shown by Sadras and Lawson (2011). Huge vegetative canopies are also considered inadequate for environments with late season drought due to imbalanced water use. Exceptions might be for accessions where large stems provide a source for carbohydrate translocation to grains. The saving types with restricted water loss and low dry matter accumulation might provide adaptive advantages under high stress environments. Still considering landraces as accessions selected for productivity under low input conditions, a constitutive restriction of growth due to conservative resource use could be a selection disadvantage. This might have reduced the presence of saving types among landraces. The allocation of cultivars toward the capacitance type confirms findings of Nakhforoosh et al. (2015) from field trials: cultivars have been selected for compact canopies that ensure sufficient light interception for photosynthesis in field stands, while high stomata opening allows an optimization of CO_2_ assimilation. Nakhforoosh et al. (2014) also revealed that such types often show effective water uptake to sustain high stomata conductance.

Also several retrospective studies on yield progress of wheat cultivars released over the last decades concluded that the ideotype of modern cultivars has short stature (Slafer and Araus, 2007), small and erect leaves (Austin et al., 1980; Feil, 1992; Fischer, 2001), restricted tillering capacity (Richards et al., 2010), and enhanced stomata functioning compensating reduced leaf area in terms of assimilation (Fischer et al., 1998; Sadras and Lawson, 2011). These characteristics confer high assimilation potential as well as high transpiration efficiency to modern cultivars such as Neda (Shearman et al., 2005). Already during early stage phenotyping modern cultivars showed distinctive canopy traits and water use type compared to most landraces. Within a breeding process for better drought resistance, the early stage phenotyping based distinction among water use strategies can contribute to narrowing a screening population while keeping diversity of the sample. Properly interpreted phenotyping data thereby provide a gate from an early stage of the breeding process toward subsequent field validation of the trait implications for yield formation in a given target drought environment.

## Conclusion

Plant phenotyping provides important trait based information to enhance the breeding process toward yield improvement and higher stress resistance. Using a shoot imaging platform (ScreenHouse) we investigated water use strategies and dry matter accumulation of durum wheat landraces from different regions of origin compared to modern cultivars. During early stage phenotyping of pot grown plants, leaf area development is the main driver of dry matter accumulation and transpiration. Growth and water use under both, optimum water supply and drought, are therefore higher in landrace accessions with extensive canopies compared to genotypes with more compact architecture. The common genotype response to a constantly lower water regime and their unchanged ranking in dry matter and transpiration suggested that differences are largely constitutive. More severe experimental stress conditions, e.g., prolonged soil drying until wilting, still might provide growth advantages to water saving accessions with reduced canopy size.

Morphological phenotyping information was combined with stomata conductance as key physiological trait. Thereby four strategies of water use and biomass growth were identified: landraces with high water use and dry matter accumulation due to large leaf area (area type), and additionally high stomata conductance (spending type). All cultivars grouped within the conductance type having reduced canopy size but optimized physiology, providing high growth potential per unit of leaf area. Beyond leaf area limitation during the early vegetative stage, productivity per unit leaf area is considered essential to ensure high yield potential. Only few accessions were water savers with both low leaf area and stomata conductance. The constitutive limitation of growth potential seems to be a negative criterion for crop performance in most agro-ecosystems, reducing water savers in the landrace genepool.

Further assessment of genotypes should make use of the identification of four distinct water use types, selecting single accessions allocated to each group. Neda best represents a conductance type with high transpiration efficiency due to strong photosynthetic capacity. The Turkish accession Ziraat on the contrary is a large leaf area type where low stomata conductance underlies high transpiration efficiency. The Ethiopian landrace ELS63 is representative for spending types with extensive canopy and high stomata conductance, suggesting the need for an efficient root system to sustain this strategy. A saving type with potential interest for high stress environments would be accession 9923 from Lebanon with low conductance and low leaf area. We conclude that the identified morphological and physiological trait combination provides an appropriate framework for targeted selection of genetic material and subsequent field testing the implications of distinct water use strategies for crop productivity in different drought environments.

## Author contributions

AN has conducted the experiment and contributed to data evaluation and writing of the manuscript. TB has contributed to the preparation and conducting of the experiment and measurements. FF has contributed to the concept of experiment and measurements of this study as well as to writing the manuscript. GB has contributed to the experimental concept, to the evaluation of data, conceptualizing, and writing the manuscript.

### Conflict of interest statement

The authors declare that the research was conducted in the absence of any commercial or financial relationships that could be construed as a potential conflict of interest.
