# Supplementary material for: Identification of Water Use Strategies at Early Growth Stages in Durum Wheat from Shoot Phenotyping and Physiological Measurements
Source: Front Plant Sci. 2016 Aug 5;7:1155. doi: 10.3389/fpls.2016.01155 (PMC4974299; doi:10.3389/fpls.2016.01155)
Supplement: Supplementary file 1 [file Supplementarymaterial.pdf]

Supplementary material

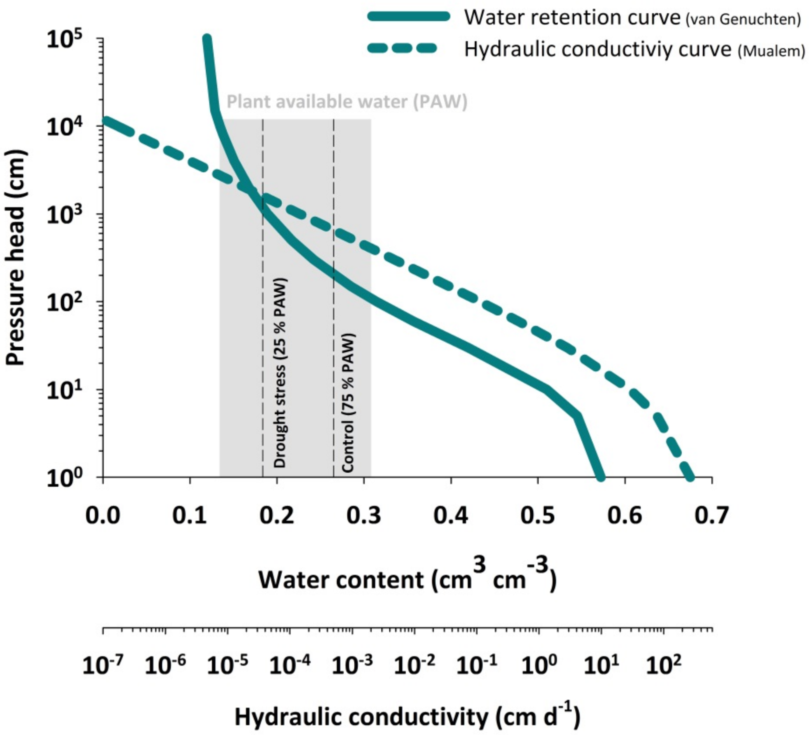

Figure S1: Water retention and hydraulic conductivity curves of the soil substrate. Shaded area represents plant available water (PAW) defined as the amount of water remained in soil between field capacity (h=-100 cm) and permanent wilting point (h=-15,000 cm). Vertical dashed lines show the volumetric soil water contents corresponding to 75 % and 25 % of the PAW.

Description of the Screen House Shoot Phenotyping System (IBG2 Plant Sciences, Forschungszentrum Jülich, Germany)

The Screen-House System is a prototype designed by the company VISSER (Visser Horti Systems, Gravendeel, Netherlands) and was completed and established by the research center Jülich, IBG2 Plant Science. The recent maximum capacity is 500 pots per day. The phenotyping system Screen-House is used for measuring shoot development and structure of different mono- and dicotyledonous plant species (e.g. rapeseed, maize, tomato, sugar beet, cereals) under different environmental scenarios. It is located in a semi-controlled compartment of the research greenhouse Phytech greenhouse at Forschungszentrum Jülich GmbH, Institute of Bio- and Geo-Sciences, IBG2 Plant Sciences, 50°55′20″N 06°21′30″E. Screen House enables continuous and non-invasive analysis of shoot properties over a period of several weeks for individual plants. Additional physiological measurements such as assessment of photosynthesis or pigment content can be performed using portable instruments providing additional information about the status of the plants.

Technical description

The Screen-House experimental setup (Figure S2) is a plant-to sensor automated system for shoot growth characterization. The system is equipped with a laser controlled positioning system (SICK Vertriebs-GmbH, Düsseldorf, Germany - DME5000-212 laser class 2, distance measure sensor) which enables a mechanical gripper to reach pre-defined positions on cultivation tables in the greenhouse and transport individual plants to the imaging station. The imaging is routinely performed with three cameras (Resolution: 2448 x 2048Px; Frame Rate: 15 FPS; 5.0 MegaPx; Sony ICX625/ CCD) located in an imaging station at three fixed different positions (180°, 90° and 45° angle).

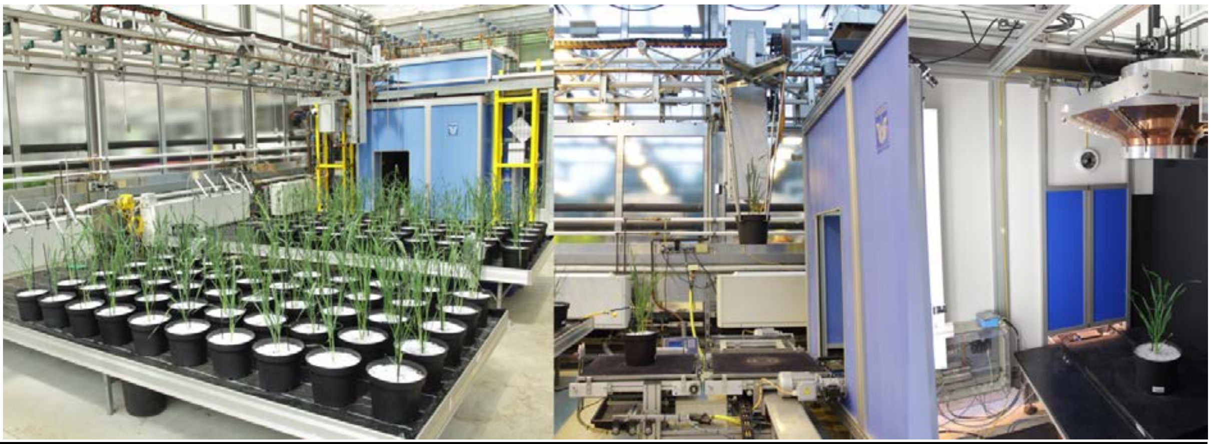

Figure S2 Left Panel: Overview of the Screen-House setup showing the cultivation tables, the positioning system (top left) and the imaging station (blue construction in the background). Middle Panel: Detail of the transporting gripper and of the weighing tablet. Right Panel: View of the imaging position on motorized rotating table. One of the three CCD cameras (top left) is visible in this picture. The plants shown in the left panel are the durum wheat genotypes that were investigated in the experiments described in the main text of this manuscript.

For the imaging routine the plants are positioned on a rotating, motorized tablet which enables exposing each to the cameras from any desired view with possible steps of 1°. The imaging station) is illuminated by one LED ring (Walimexpro-Mediaresort, Altena Germany - LED ring light) and 6 halogen lamps (Osram GmbH, München Germany – Lumix Cool White L36W) for ensuring homogenous light conditions. Additionally, the Screen-House

experimental setup is equipped with a balance (Figure S2, middle B, Mettler Toledo, Gießen Germany – SSP1241) for automated gravimetric measurements of plants grown in pots.

Image Processing Pipeline

The image acquisition and processing pipeline is consisting of the different steps which are shown in the process diagram (Figure S3). In the image acquisition process of the Screen-House system the pictures are stored as a raw format (grayscale Bayer format). This format contains only the information of brightness. RGB color values at each image position I(x,y) are obtained by interpolating each pixel with its surrounding neighbors.

The next step of the image processing pipeline is the undistortion of the pictures. Every camera lens has a unique distortion model based on the parameter of the camera and the optic. This distortion can be corrected by calibrating each camera with a checkerboard target. The information of the distortion model for each Screen-House camera are stored in text files and passed to the image processing pipeline.

To make the following segmentation procedure more robust, we integrated the option of a background subtraction in the segmentation pipeline. For this operation, an image B of the empty measurement chamber was taken for every camera and subtracted from each corresponding image I: $S(x,y) = I(x,y) - B(x,y)$. Because of small changes in the spatial domain of the image caused by movement coming from vibrations or sensor noise, each image I and B was blurred with a 5x5 Gaussian kernel before the subtraction.

Each pixel in S with intensity above a user defined threshold is considered as consistent in the image setup and set to zero. This operation can be used to remove fixed objects like cables or other installations before segmentation, but not objects like pots, so a final segmentation is still needed.

After the undistortion of the pictures the actual image segmentation, i.e., the separation of the plant from the background is performed. The segmentation method we use is based on Support Vector Machines (SVM) using features from RGB or HSV color space. The SVM classifier is trained once before segmentation with separate training software. This software takes example images for fore- and back-ground as input and extracts the relevant features in the chosen color channels. The training information of the SVM classifier can be stored in xml files and passed to the image processing pipeline.

After the segmentation small artifacts and holes in the obtained mask where removed by using connected-component labeling. Based on these binary masks, several plant traits like projected leaf area, plant height or mean color values where calculated by the pipeline. All calculated values were stored in a final CSV file including metadata like Plant ID, image acquisition time and camera.

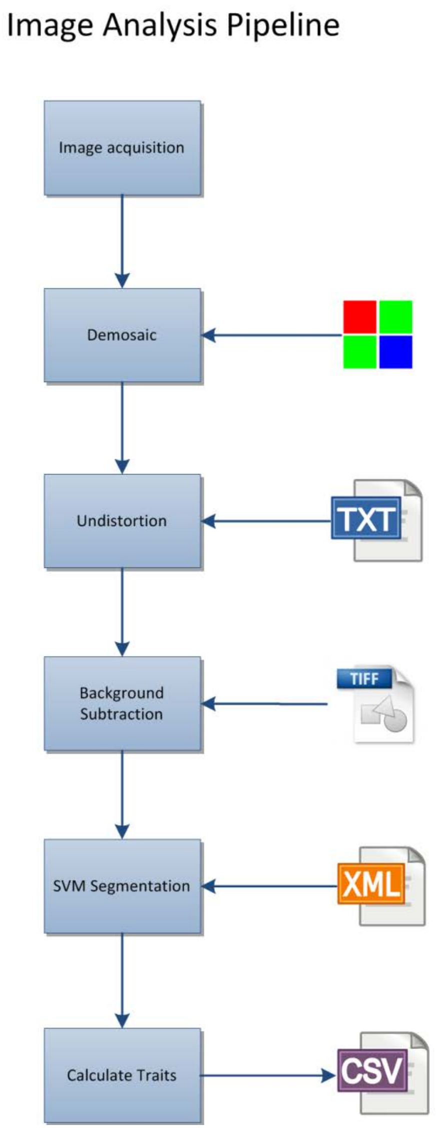

Figure S3 Process diagram of the image acquisition and processing for digital pictures of plant shoots in the SCREEN-House phenotyping imaging system.

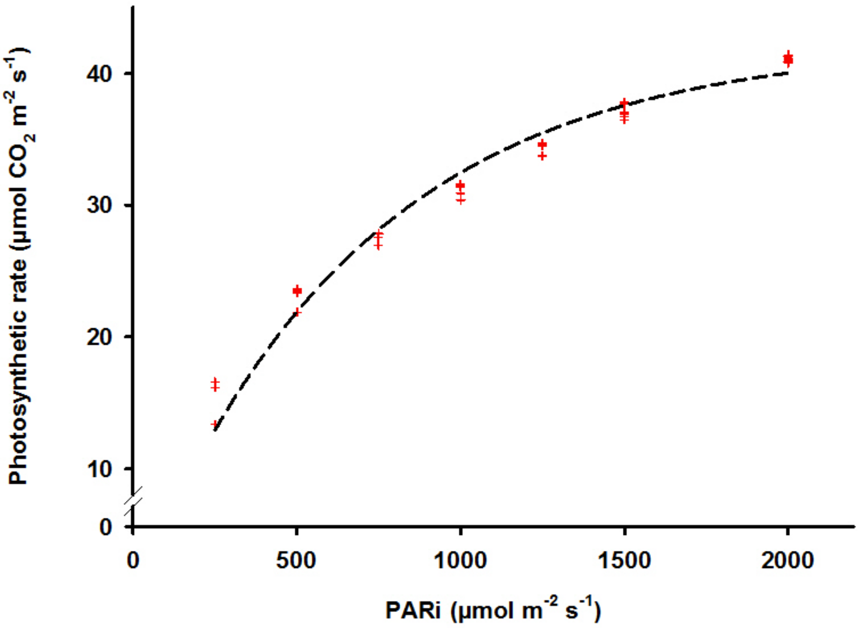

Figure S4: Light response curve of cultivar Floradur to determine saturating light intensity to be used for measurement of maximum photosynthetic rate.

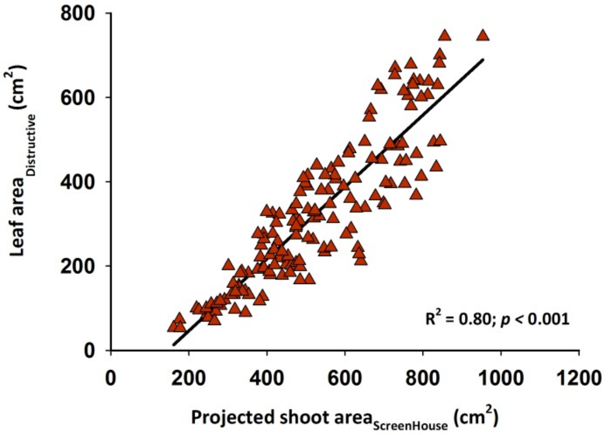

Figure S5: Association between image based projected shoot area from ScreenHouse and destructively measured leaf area after harvesting.
